# Global, regional, and national trends in gastric cancer burden: 1990-2021 and projections to 2040

**DOI:** 10.3389/fonc.2024.1468488

**Published:** 2024-12-12

**Authors:** Tao Zhang, Yiqun Zhang, Xiaofei Leng

**Affiliations:** ^1^ Department of Gastric and Colorectal Surgery, General Surgery Center, The First Hospital of Jilin University, Changchun, Jilin, China; ^2^ Department of Gynecology, Taihe Hospital, Hubei University of Medicine, Shiyan, Hubei, China; ^3^ State Key Laboratory of Ultrasound in Medicine and Engineering, Chongqing Medical University, Chongqing, China; ^4^ Department of Gynecologic Oncology, Beijing Obstetrics and Gynecology Hospital, Capital Medical University, Beijing, China

**Keywords:** gastric cancer, global burden of disease, incidence, mortality, disability-adjusted life years

## Abstract

**Background:**

Gastric cancer (GC) is a common malignancy of the digestive system, with significant geographical variation in its disease burden.

**Methods:**

This study used data from the Global Burden of Diseases, Injuries, and Risk Factors Study 2021 to analyze three key indicators: incidence, mortality, and disability-adjusted life years (DALYs). Initially, a detailed analysis of the GC burden was conducted from global, regional, national, gender, and age perspectives. Subsequently, the percentage change and average annual percent change (AAPC) of GC were calculated to understand the trends in disease burden. Decomposition analysis and frontier analysis were then performed. Finally, the Bayesian age-period-cohort model was used to predict the trends in age-standardized rates (ASRs) of GC up to 2040.

**Results:**

In 2021, there were 1.23 million (95% UI: 1.05-1.41 million) new cases of GC globally, with 0.95 million (95% UI: 0.82-1.10million) deaths and 22.79 million (95% UI: 19.58-26.12 million) DALYs. Compared to 1990, the global ASRs of GC has declined, but new cases and deaths have increased. For females, age-standardized incidence rate, age-standardized mortality rate, and age-standardized DALYs rate were 8.6, 7.1, and 165.6 per 100,000, with AAPCs of -2.1, -2.4, and -2.6. For males, they were 20.9, 16.0, and 371.2 per 100,000, with AAPCs of -1.6, -2.1, and -2.3. ASRs fluctuated with increasing Socio-demographic Index (SDI), being higher in middle and high-middle SDI regions. Decomposition analysis indicated negative effects from epidemiological trends on GC burden, while population growth and aging had positive effects. Frontier analysis showed that middle and high-middle SDI regions had more potential for reducing ASRs. Predictions indicate a continued decline in ASRs for both genders by 2040.

**Conclusion:**

Despite progress in controlling GC, the number of new cases and deaths globally is rising due to population growth and aging. This highlights the need for effective prevention and control strategies.

## 1 Introduction

Gastric cancer (GC) is a common and highly lethal malignancy of the digestive system (1). Although the age-standardized incidence rate (ASIR) and age-standardized mortality rate (ASMR) of GC have declined over the past century, it remains a significant global health burden (2). According to the latest statistics from GLOBOCAN 2022, GC ranks fifth in both incidence and mortality among cancers (3). The epidemiology of GC shows significant geographic and gender differences (4). Studies indicate that East Asia, High-income Asia Pacific, and South Asia have the highest incident cases, deaths, and disability-adjusted life years (DALYs), while Oceania, Australasia, and sub-Saharan Africa have the lowest (5).

Due to population aging and growth, the number of GC cases is expected to increase in the future (5). Additionally, early-stage GC patients often exhibit no obvious symptoms, and the disease is typically diagnosed at a late stage (6, 7). At diagnosis, common symptoms include anorexia, indigestion, weight loss, and abdominal pain (6). Despite recent improvements in survival rates, the five-year survival rate for GC remains low (2). Therefore, understanding the global distribution and epidemiological trends of GC is crucial for developing public health policies tailored to different regions to reduce the disease burden.

Our study utilizes the latest data released by the Global Burden of Diseases, Injuries, and Risk Factors Study (GBD) 2021 in May 2024, analyzing incidence, mortality, and DALYs to systematically evaluate the distribution differences of GC across various regions and countries globally, and the changing trends in the burden from 1990 to 2021 (8–10). Additionally, we used the Bayesian Age-Period-Cohort (BAPC) model to project age-standardized rates (ASRs) of GC to 2040. The results of this study will aid in understanding the dynamic changes in the global and regional burden of GC and provide a reference for developing targeted prevention and control strategies.

## 2 Materials and methods

### 2.1 Data sources

The GBD 2021 dataset encompasses 371 diseases and injuries, offering annual estimates from 1990 to 2021 for metrics such as incidence, mortality, and DALYs across 204 countries and territories (8–10). Data are disaggregated by gender (both sex, female and male) and age. This comprehensive dataset is provided by the Institute for Health Metrics and Evaluation at the University of Washington and is available for free download from https://vizhub.healthdata.org/gbd-results/. The DALYs estimates for GBD 2021 draw from a total of 100,983 data sources, including 19,189 newly used in 2021 (10). Among these, 75,459 data sources focused on non-fatal conditions, including 36,916 on incidence, 22,236 on prevalence, and 45 on other epidemiological measures. Non-fatal estimates derive from sources such as scientific literature, household surveys, epidemiological surveillance data, disease registries, clinical informatics, and other sources. Mortality and years of life lost estimates are based on 56,604 data sources, which include vital registrations, verbal autopsies, and data from surveys, censuses, surveillance systems, and cancer registries (8). As in previous GBD cycles, the majority of cause-specific mortality rates are estimated using the Cause of Death Ensemble model, a tool developed by GBD to assess the predictive validity of various statistical models and covariate combinations, synthesizing these results to generate mortality estimates. For causes with limited data, substantial changes over time, or unusual epidemiological patterns, alternative modeling strategies were applied. GBD 2021 continues to define diseases based on the International Classification of Diseases codes, as detailed in the original GBD literature. In this study, we focused on the incidence, mortality, and DALYs for GC, utilizing data from global sources, covering 204 countries and regions, 21 GBD regions, 4 continents, and 5 Socio-demographic Index (SDI) groupings.

### 2.2 Statistical analysis

Data on new cases from 1990 and 2021 were obtained from GBD 2021, covering global estimates (by all sexes, male, and female), 21 GBD regions, five SDI groups, and 204 countries and territories. The percentage change in new cases was calculated using the formula: (new cases in 2021 - new cases in 1990)/new cases in 1990 × 100% (11). This same approach was applied to calculate the percentage change in GC deaths and DALYs from 1990 to 2021. Additionally, ASIR, ASMR, and age-standardized DALYs rate for GC from 1990 to 2021 were gathered for these regions. The standard error (SE) was calculated using the formula: SE = (upper - lower)/(1.96 × 2), where upper and lower denote the upper and lower bounds of the uncertainty interval (UI) for ASRs provided by GBD 2021 (12). The joinpoint regression analysis was then conducted using the Desktop version of the joinpoint software from the National Cancer Institute (https://surveillance.cancer.gov/joinpoint/) (13, 14). In this analysis, the Permutation Test was applied, and the confidence intervals (CIs) for APC and AAPC were calculated using the Parametric Method, with other settings kept as the software defaults.

This study examined the trend of ASRs for GC as SDI increases. Initially, a scatter plot was created using the ggplot2 (3.5.1) package in R software, with SDI on the x-axis and ASRs on the y-axis. The geom_smooth function from ggplot2 was then used for curve fitting, with the following settings: color = “black,” stat = “smooth,” method = “loess,” se = FALSE, and span = 0.5; other parameters were kept at their default values.

Using decomposition analysis based on the Das Gupta method, we visually illustrated the contributions of population growth, epidemiological trends, and population aging to changes in incident cases, deaths, and DALYs (15, 16). Frontier analysis is a method for evaluating and comparing the efficiency and performance of different countries and territories in health metrics (17, 18). In our study, we calculated the lower limit of the ASR that each country and territories could achieve at the current SDI level, comparing the current ASRs to this lower limit to assess their performance levels.

BAPC projection is an analytical technique based on Bayesian statistics, used to predict future events or trends, particularly in the health and social sciences (19, 20). BAPC projection effectively decomposes and estimates age, period, and cohort effects, providing future event predictions and quantifying uncertainty, and can flexibly handle complex data structures. In our study, we used the BAPC (0.0.36) and INLA (24.6.27) packages to fit the model and project trends in ASRs to 2040 (19–21).

A more detailed description of the above analytical methods is provided in the **Supplementary Methods** of this study. The data used in our study are from publicly available databases, freely accessible for download, and have received ethical approval in their original studies, thus requiring no additional ethical clearance. The joinpoint regression analysis in this study was performed using the Joinpoint software (5.2.0.0), while all other analyses were conducted in R software (4.4.1). The world map in this study was created using the map_data function from the ggplot2 (3.5.1) package. This study strictly adhered to the Strengthening the Reporting of Cohort, Cross-Sectional and Case-Control Studies in Surgery criteria (**Supplementary Table 1**) (22).

## 3 Results

### 3.1 Global Burden of GC

In 2021, the estimated number of new GC cases worldwide was 1.23 million (95% UI: 1.05–1.41 million), a 25.4% (95% UI: 11.1–42.2) increase compared to 1990 (**Table 1**). The global ASIR was 14.3 per 100,000 population (95% UI: 12.2–16.4), showing an overall declining trend (AAPC: -1.8, 95% CI: -1.9 to -1.6) (**Table 1**). The number of GC deaths was 954,000 (95% UI: 822,000–1,090,000), an 11.7% (95% UI: -0.7–26.8) increase from 1990, with an ASMR of 11.2 per 100,000 (95% UI: 9.6–12.7), also showing a declining trend (AAPC: -2.2, 95% CI: -2.3 to -2.1) (**Supplementary Table 2**). In 2021, the global DALYs due to GC reached 22.79 million (95% UI: 19.58–26.12 million), a 1.9% (95% UI: -13.0–11.9) decrease from 1990, with an age-standardized DALYs rate of 262.7 per 100,000 (95% UI: 226.1–301.0), continuing a downward trend (AAPC: -2.4, 95% CI: -2.5 to -2.3) (**Supplementary Table 3**).

**Table 1:** Incident cases and ASIR of GC in 1990 and 2021, by gender and GBD region.

Characteristics	Incident cases			ASIR		
	1990 (95% UI)	2021 (95% UI)	Percentage change (%, 95% UI)	1990 (95% UI)	2021 (95% UI)	AAPC (95% CI)
Global	981,000 (891,000–1,070,000)	1,230,000 (1,050,000–1,410,000)	25.4 (11.1–42.2)	24.8 (22.6–27.0)	14.3 (12.2–16.4)	-1.8 (-1.9 to -1.6)*
Sex						
Female	353,000 (322,000–385,000)	397,000 (344,000–447,000)	12.6 (-0.8–28.2)	16.6 (15.1–18.1)	8.6 (7.5–9.7)	-2.1 (-2.2 to -2.0)*
Male	628,000 (541,000–706,000)	833,000 (688,000–1,010,000)	32.6 (11.8–57.7)	34.4 (30.0–38.7)	20.9 (17.2–25.2)	-1.6 (-1.7 to -1.5)*
21 GBD Regions						
East Asia	416,000 (345,000–486,000)	625,000 (484,000–779,000)	50.3 (19.4–96.0)	47.1 (39.5–55.5)	28.6 (22.2–35.5)	-1.6 (-1.7 to -1.5)*
Southeast Asia	28,000 (22,600–32,100)	47,800 (41,100–56,400)	70.4 (47.5–109.0)	10.7 (8.7–12.3)	7.3 (6.3–8.7)	-1.2 (-1.3 to -1.2)*
South Asia	46,700 (39,900–59,200)	84,800 (73,700–102,000)	81.5 (58.0–112.6)	7.7 (6.6–9.9)	5.7 (4.9–6.8)	-1.0 (-1.3 to -0.7)*
Central Asia	12,600 (12,000–13,300)	9,760 (8,800–10,900)	-22.5 (-30.5 to -14.1)	26.4 (25.1–27.8)	11.8 (10.7–13.1)	-2.6 (-2.8 to -2.5)*
Eastern Europe	92,700 (90,200–94,600)	51,000 (46,900–55,000)	-45.0 (-49.6 to -40.4)	32.9 (32.0–33.6)	14.7 (13.5–15.8)	-2.6 (-3.0 to -2.2)*
Central Europe	27,300 (26,200–28,200)	20,300 (18,700–21,900)	-25.7 (-31.0 to -19.4)	18.3 (17.6–19.0)	9.2 (8.5–9.9)	-2.2 (-2.3 to -2.1)*
Western Europe	99,500 (93,600–103,000)	73,900 (65,900–78,800)	-25.7 (-30.4 to -21.6)	17.0 (16.0–17.6)	7.8 (7.1–8.3)	-2.5 (-2.6 to -2.4)*
North Africa and Middle East	24,700 (18,600–27,900)	42,600 (29,700–48,500)	72.3 (51.6–97.5)	14.7 (11.1–16.6)	9.7 (6.8–10.9)	-1.3 (-1.4 to -1.2)*
Eastern Sub-Saharan Africa	8,230 (6,530–9,430)	11,600 (9,550–13,700)	41.5 (23.6–68.7)	10.7 (8.5–12.1)	6.8 (5.7–7.9)	-1.5 (-1.5 to -1.4)*
Western Sub-Saharan Africa	6,580 (5,590–7,730)	12,100 (9,340–14,200)	83.5 (51.0–116.8)	7.6 (6.5–9.0)	6.3 (5.0–7.5)	-0.6 (-0.6 to -0.5)*
Southern Sub-Saharan Africa	2,180 (1,710–2,440)	4,050 (3,380–4,570)	86.2 (65.0–110.3)	7.9 (6.2–8.9)	7.0 (5.8–7.9)	-0.4 (-0.8 to -0.0)*
Central Sub-Saharan Africa	2,570 (1,890–3,120)	4,660 (3,480–5,780)	81.6 (38.1–138.7)	11.5 (8.6–13.9)	8.5 (6.4–10.5)	-1.0 (-1.2 to -0.8)*
Central Latin America	16,300 (15,700–16,800)	29,800 (26,600–33,500)	83.0 (63.9–105.0)	20.2 (19.3–20.8)	12.0 (10.7–13.4)	-1.7 (-1.9 to -1.5)*
Southern Latin America	8,560 (8,080–9,060)	9,970 (9,010–10,800)	16.4 (6.2–27.6)	18.7 (17.6–19.8)	11.4 (10.3–12.4)	-1.6 (-1.9 to -1.3)*
Andean Latin America	6,290 (5,530–7,160)	12,600 (10,200–15,400)	100.3 (61.2–151.7)	31.1 (27.4–35.5)	21.5 (17.4–26.3)	-1.1 (-1.9 to -0.4)*
Tropical Latin America	16,900 (16,100–17,500)	25,600 (23,900–26,900)	51.9 (44.6–59.0)	18.9 (17.9–19.7)	10.0 (9.3–10.5)	-2.0 (-2.3 to -1.8)*
Caribbean	3,130 (2,870–3,440)	4,280 (3,710–4,900)	36.6 (18.5–57.0)	12.2 (11.1–13.3)	8.0 (6.9–9.1)	-1.3 (-1.6 to -1.0)*
Australasia	2,410 (2,260–2,540)	3,190 (2,820–3,480)	32.3 (20.2–44.9)	10.3 (9.6–10.8)	5.9 (5.3–6.4)	-1.8 (-2.2 to -1.4)*
Oceania	513 (364–662)	1,010 (787–1,260)	96.8 (49.4–152.0)	17.1 (12.6–21.8)	13.3 (10.5–16.3)	-0.8 (-0.9 to -0.8)*
High-income Asia Pacific	131,000 (124,000–136,000)	123,000 (107,000–133,000)	-5.8 (-15.0–2.3)	65.2 (61.4–67.7)	25.4 (22.8–27.3)	-3.0 (-3.2 to -2.8)*
High-income North America	29,200 (27,500–30,200)	33,100 (30,800–34,700)	13.2 (9.1–17.0)	8.3 (7.8–8.6)	5.2 (4.9–5.5)	-1.5 (-1.6 to -1.4)*
5 SDI Regions						
High SDI	254,000 (242,000–262,000)	239,000 (215,000–257,000)	-6.0 (-12.4 to -0.8)	23.1 (22.0–23.8)	11.2 (10.2–11.9)	-2.3 (-2.5 to -2.2)*
High-middle SDI	333,000 (298,000–361,000)	387,000 (316,000–457,000)	16.4 (-0.7–38.7)	33.3 (30.1–36.1)	19.6 (16.0–23.1)	-1.7 (-1.8 to -1.6)*
Middle SDI	304,000 (262,000–351,000)	451,000 (368,000–542,000)	48.6 (23.4–81.1)	28.9 (25.1–33.4)	16.9 (13.8–20.3)	-1.7 (-1.9 to -1.6)*
Low-middle SDI	63,100 (55,300–76,800)	110,000 (97,100–126,000)	75.0 (57.4–98.0)	10.1 (9.0–12.4)	7.7 (6.7–8.8)	-0.9 (-1.0 to -0.7)*
Low SDI	26,300 (21,000–30,100)	41,400 (32,600–47,000)	57.2 (40.4–81.0)	11.4 (9.0–13.0)	8.1 (6.4–9.2)	-1.1 (-1.2 to -1.0)*

Statistically significant AAPC results are marked with an asterisk (*). ASIR, age-standardized incidence rate; GC, gastric cancer; GBD, Global Burden of Diseases, Injuries, and Risk Factors Study; UI, uncertainty interval; AAPC, average annual percentage change; CI, confidence interval; SDI, Socio-demographic Index.

### 3.2 GC burden by GBD region and SDI level

In the 21 GBD regions, East Asia (624,688.18 cases;. 95% UI: 483569.21–778628.95) and High-income Asia Pacific (123,000 cases; 95% UI: 107,000–133,000) had the highest number of GC cases in 2021, followed by South Asia (85,000 cases; 95% UI: 74,000–102,000) and Western Europe (74,000 cases; 95% UI: 66,000–79,000) (**Table 1**). Notably, East Asia accounted for over half of the global GC cases. Compared to 1990, Andean Latin America, Oceania, Southern Sub-Saharan Africa, and Western Sub-Saharan Africa showed the largest increases in the number of cases (**Table 1**). In terms of ASIR, East Asia (28.6 per 100,000; 95% UI: 22.2–35.5) and High-income Asia Pacific (25.4 per 100,000; 95% UI: 22.8–27.3) were at the highest levels globally, while South Asia (5.7 per 100,000; 95% UI: 4.9–6.8) and Western Europe (7.8 per 100,000; 95% UI: 7.1–8.3) had relatively lower ASIRs (**Table 1**). Significant ASIR increases were observed in Southern Sub-Saharan Africa, Western Sub-Saharan Africa, Oceania, and South Asia (**Table 1**).

Similarly, the regions with the highest numbers of deaths were East Asia (456,000 deaths; 95% UI: 356,000–567,000), South Asia (83,000 deaths; 95% UI: 71,000–99,000), High-income Asia Pacific (71,000 deaths; 95% UI: 60,000–77,000), and Western Europe (56,000 deaths; 95% UI: 50,000–60,000) (**Supplementary Table 2**). Oceania, Andean Latin America, Southern Sub-Saharan Africa, and Western Sub-Saharan Africa saw the largest increases in deaths (**Supplementary Table 2**). East Asia (21.3 per 100,000; 95% UI: 16.6–26.2) and High-income Asia Pacific (13.1 per 100,000; 95% UI: 11.5–14.1) maintained high ASMR, with Andean Latin America (21.3 per 100,000; 95% UI: 17.3–26.1) at the highest level. The most substantial ASMR increases were observed in Southern Sub-Saharan Africa, Western Sub-Saharan Africa, Oceania, and South Asia (**Supplementary Table 2**).

In 2021, the highest DALYs from GC were recorded in East Asia (10.92 million; 95% UI: 8.49–13.67 million), followed by South Asia (2.30 million; 95% UI: 2.00–2.77 million), High-income Asia Pacific (1.19 million; 95% UI: 1.06–1.29 million), and Southeast Asia (1.19 million; 95% UI: 1.02–1.40 million) (**Supplementary Table 3**). The regions with the largest increases in DALYs were Oceania, Western Sub-Saharan Africa, Central Sub-Saharan Africa, and Southern Sub-Saharan Africa (**Supplementary Table 3**). Age-standardized DALYs rates were highest in East Asia (496.9 per 100,000; 95 UI: 386.2–619.7), Andean Latin America (485.4 per 100,000; 95% UI: 391.2–595.1), Oceania (341.8 per 100,000; 95% UI: 265.8–428.0), and Eastern Europe (306.6 per 100,000; 95% UI: 280.0–332.6), with the greatest increases observed in Southern Sub-Saharan Africa, Western Sub-Saharan Africa, Oceania, and Central Sub-Saharan Africa (**Supplementary Table 3**).

**Figure 1:**
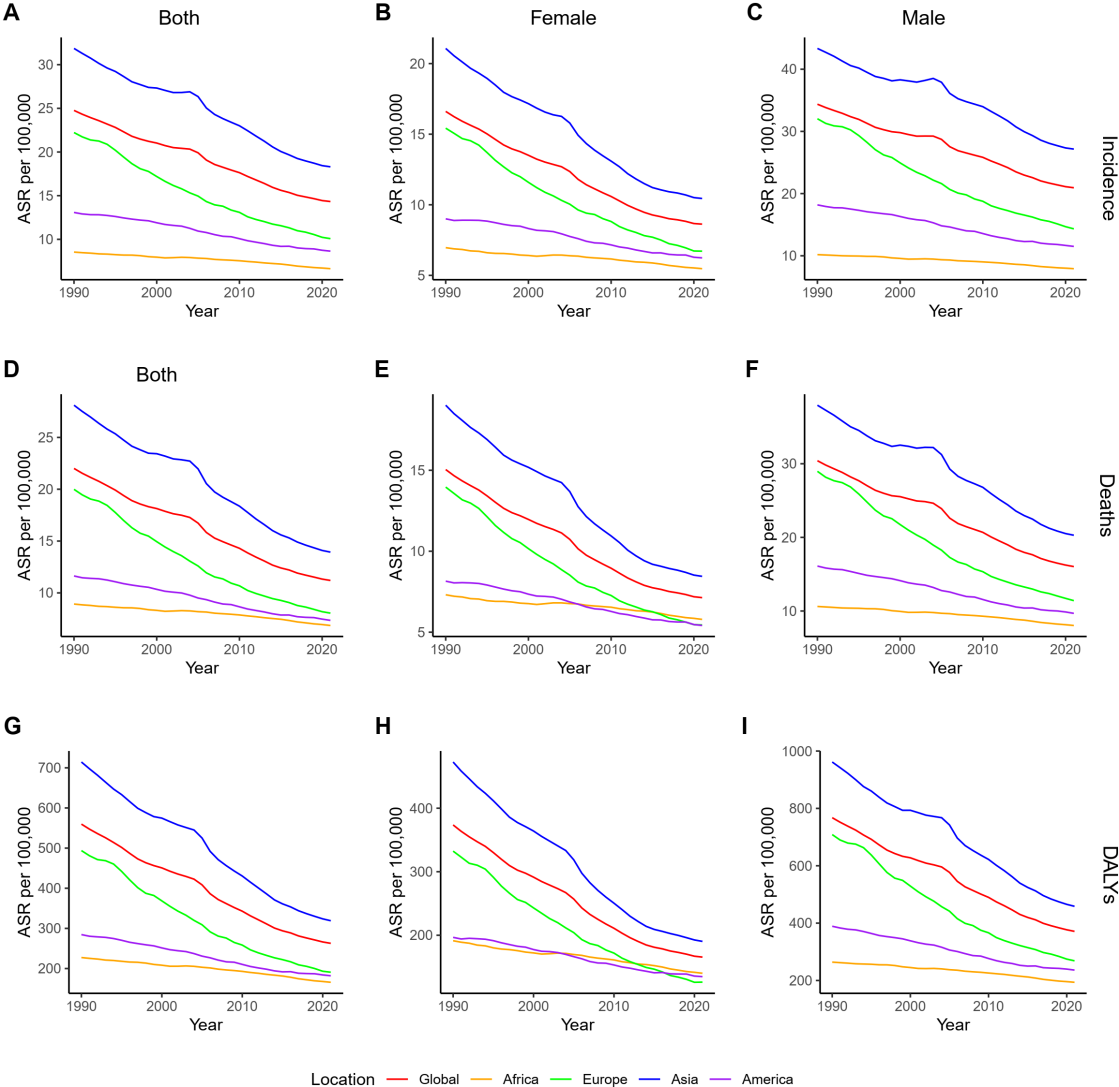
ASRs of GC by gender across continents from 1990 to 2021. **(A–C)** ASIR for both sexes, female, and male; **(D–F)** ASMR for both sexes, female, and male; **(G–I)** age-standardized DALYs rate for both sexes, female, and male. ASR, age-standardized rate; GC, gastric cancer; DALYs, disability-adjusted life years. ASIR, age-standardized incidence rate; ASMR, age-standardized mortality rate.

**Figure 2:**
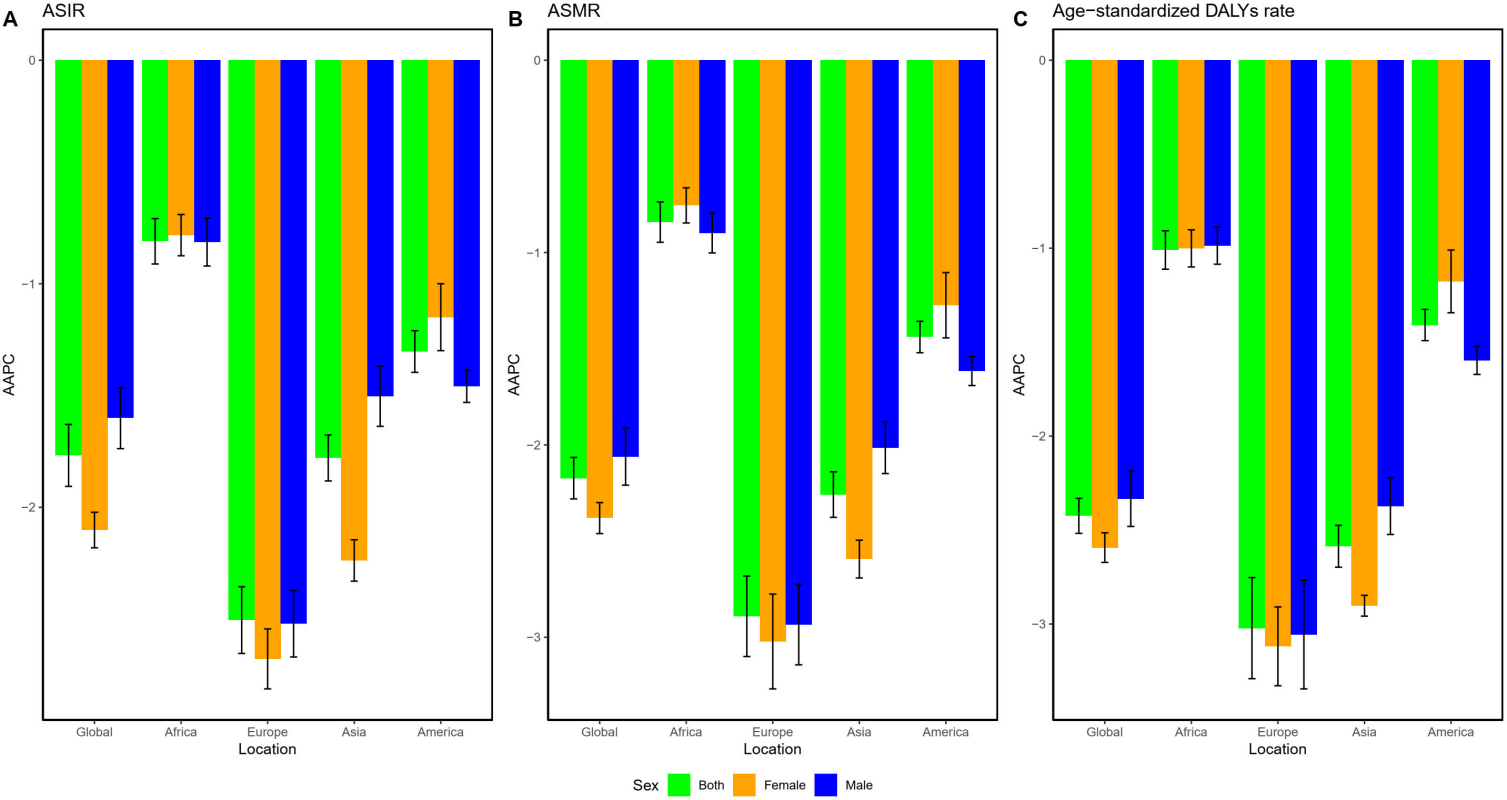
AAPC of GC by gender across continents from 1990 to 2021. **(A)** ASIR; **(B)** ASMR; **(C)** age-standardized DALYs rate. AAPC, average annual percentage change; GC, gastric cancer; DALYs, disability-adjusted life years; ASMR, age-standardized mortality rate; ASIR, age-standardized incidence rate.

In the analysis across continents, the ASRs showed a declining trend from 1990 to 2021 in four major continents (**Figures 1**, **2**). Among them, the decline in ASRs of GC is the largest in Europe and the smallest in Africa (**Figure 2**). Asia and Europe had the highest ASRs, followed by the Americas and Africa, with only Asia’s ASR exceeding the global level (**Figure 1**).

**Figure 3:**
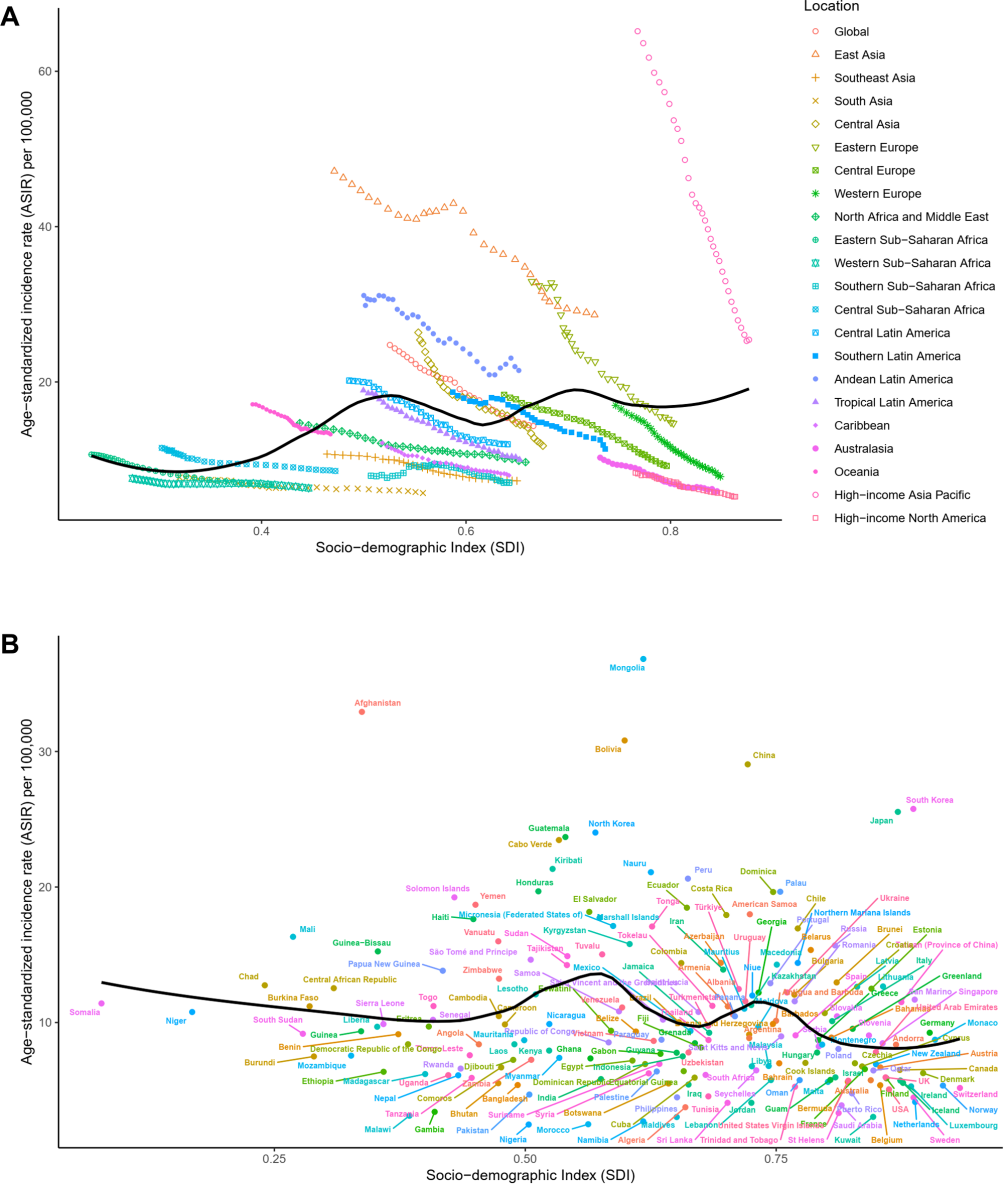
Distribution of ASIR for GC across different SDI levels. **(A)** Global and 21 GBD regions from 1990 to 2021; **(B)** 204 countries and territories in 2021. ASIR, age-standardized incidence rate; GC, gastric cancer; SDI, Socio-demographic Index; GBD, Global Burden of Diseases, Injuries, and Risk Factors Study.

Countries and territories were grouped into five categories based on SDI levels. In 2021, the middle SDI group had the highest levels of new cases, deaths, and DALYs, while the low SDI group had the lowest (**Tables 1**; **Supplementary Tables 2, 3**). From 1990 to 2021, the low-middle SDI group saw the largest increases in new cases, deaths, and DALYs, while the high SDI group showed the greatest decreases (**Tables 1**; **Supplementary Tables 2, 3**). In terms of ASRs, the high-middle SDI group had the highest ASRs in 2021, while the high SDI group exhibited the largest decrease in ASRs (**Tables 1**; **Supplementary Tables 2, 3**). In analyzing the relationship between SDI and GC ASRs, we observed a fluctuating trend: as SDI increased, ASRs initially rose, then fell, rose again, decreased once more, and finally increased again (**Figure 3**; **Supplementary Figures 1, 2**).

### 3.3 GC burden by country and territory

**Figure 4:**
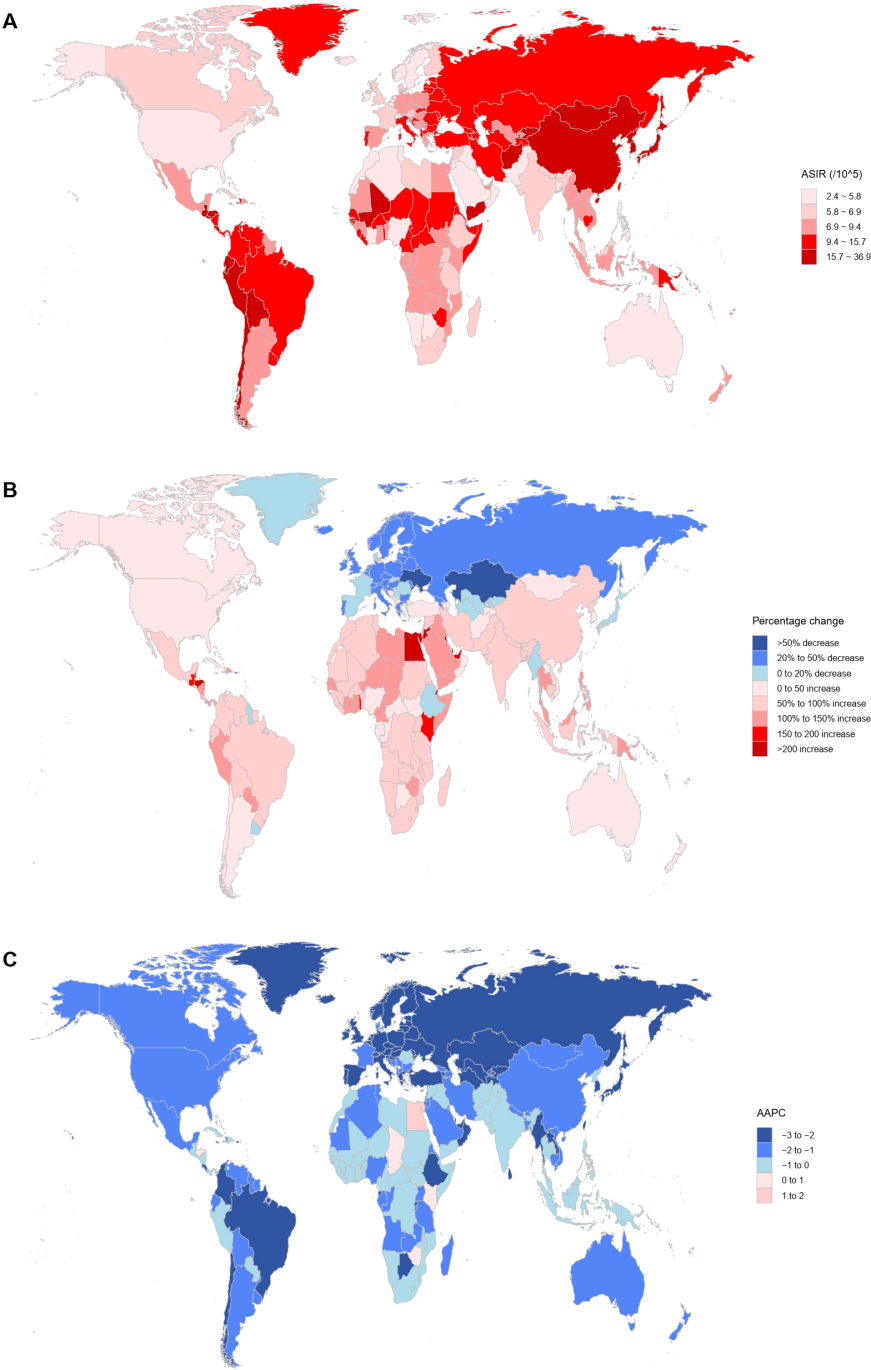
Analysis of GC incidence using multiple indicators in 204 countries and territories. **(A)** ASIR per 100,000 population in 2021; **(B)** Percentage change in the number of incident cases from 1990 to 2021; **(C)** AAPC from 1990 to 2021. GC, gastric cancer; ASIR, age-standardized incidence rate; AAPC, average annual percentage change.

In 2021, China (mainland) had 612,000 (95% UI: 472,000–766,000) new GC cases, accounting for 49.8% of the global total, making it the country with the highest number of new cases, followed by Japan (99,000 cases, 95% UI: 85,000–107,000), India (70,000 cases; 95% UI: 61,000–87,000), and Russia (37,000 cases; 95% UI: 34,000–40,000) (**Supplementary Table 4**). Compared to 1990, Djibouti (280.7%; 95% UI: 167.2–454.5), Qatar (262.9%; 152.7–408.4), and the United Arab Emirates (258.5%; 95% UI: 180.2–358.2) showed the largest increases in new cases (**Supplementary Table 4**; **Figure 4**). In terms of ASIR, China (mainland) (29.1 per 100,000; 95% UI: 22.4–36.2), South Korea (25.8 per 100,000; 95% UI: 21.5–32.4), and Japan (25.54 per 100,000; 95% UI: 23.0–27.0) were among the highest, ranking fourth, fifth, and sixth globally (**Supplementary Table 4**; **Figure 4**). The countries with the largest ASIR increases were Egypt (AAPC: 1.7, 95% CI: 1.0–2.3), Lesotho (AAPC: 1.0, 95% CI: 0.7–1.4), and Honduras (AAPC: 0.5, 95% CI: 0.1–1.0) (**Supplementary Table 4**; **Figure 4**).

In 2021, China (mainland) also had the highest number of GC deaths (445,000; 95% UI: 345,000–556,000), followed by India (69,000; 95% UI: 59,000–84,000), Japan (58,000; 95% UI: 49,000–63,000), and Russia (31,000; 95% UI: 29,000–34,000), with China’s (mainland) deaths accounting for 46.6% of the global total (**Supplementary Table 5**). Compared to 1990, the largest increases in deaths were seen in Djibouti (279.1%, 95% UI: 167.4–455.9), Egypt (245.9%, 95% UI: 67.9–367.9), and Honduras (244.66%, 95% UI: 168.4–351.6) (**Supplementary Table 5**; **Supplementary Figure 3**). The highest ASMR were in Mongolia (37.4 per 100,000; 95% UI: 29.4–45.9), Afghanistan (34.6 per 100,000; 95% UI: 19.0–46.9), and Bolivia (32.4 per 100,000; 95% UI: 24.5–42.2), with the largest ASMR increases in Egypt (AAPC: 1.6, 95% CI: 0.9–2.2), Lesotho (AAPC: 1.0, 95% CI: 0.6–1.4), and Honduras (AAPC: 0.6, 95% CI: 0.2–0.9) (**Supplementary Table 5**, **Supplementary Figure 3**).

In terms of GC DALYs in 2021, the highest numbers were in China (mainland) (10.64 million; 95% UI: 8.22–13.38 million), India (1.89 million; 95% UI: 1.65–2.35 million), Japan (925,000, 95% UI: 816,000–984,000), and Russia 738,000 (95% UI: 675,000–800,000), but these countries showed no substantial increases compared to 1990 (**Supplementary Table 6**). The countries with the largest increases in DALYs were Djibouti (263.0%, 95% UI: 146.8–439.5), the United Arab Emirates (225.8%, 95% UI: 153.0–322.3), and Egypt (213.6%, 95% UI: 60.1–321.3) (**Supplementary Table 6**, **Supplementary Figure 4**). The highest age-standardized DALYs rates were observed in Mongolia (930.4 per 100,000; 95% UI: 747.5–1,157.9), Afghanistan (910.2 per 100,000; 95% UI: 498.9–1,256.8), and Bolivia (714.4 per 100,000; 95% UI: 533.7–949.6), with Egypt (AAPC: 1.2, 95% CI: 0.5–1.9), Lesotho (AAPC: 1.2, 95% CI: 0.7–1.6), and Zimbabwe (AAPC: 0.7, 95% CI: 0.4–1.0) showing the largest increases (**Supplementary Table 6**, **Supplementary Figure 4**).

The results of the APC calculated from the joinpoint regression analysis for the global (all genders, female, and male), 21 GBD regions, 5 SDI groups, and 204 countries or territories are provided in **Supplementary Table 7**.

### 3.4 GC burden by gender and age

In 2021, the number of new GC cases in men was 2.1 times that in women. Excluding those aged 90 and above, men had higher new case numbers and incidence rates than women across all age groups. With increasing age, the number of new cases initially rose, peaking at ages 70–74, before declining, with the majority of cases concentrated in the 65–74 age group. Among men and in the overall population, GC incidence showed an initial increase followed by a decline, whereas in women, incidence continued to rise with age.

In 2021, the number of GC deaths in men was 1.89 times that in women. Excluding those aged 90 and above, men had higher deaths and mortality rates than women across all age groups. The number of deaths rose with age, peaking at 70–74 years, and was mostly concentrated in the 65–74 age range. Among men, mortality rates initially increased and then declined with age, while in the overall population and among women, mortality rates continued to increase with age.

In 2021, the DALYs due to GC in men were 2.03 times those in women. Excluding those over 90, men had higher DALYs numbers and rates than women across all age groups. With increasing age, DALYs initially increased, peaking at ages 65–69, before declining, with the highest burden observed in the 55–74 age range. In men and the overall population, DALY rates showed an initial rise followed by a decline with age, whereas in women, DALY rates continuously increased. The detailed information described above can be found in **Table 1**; **Supplementary Tables 2, 3**, **Supplementary Figure 5**.

### 3.5 Decomposition and frontier analysis of GC burden

**Figure 5:**
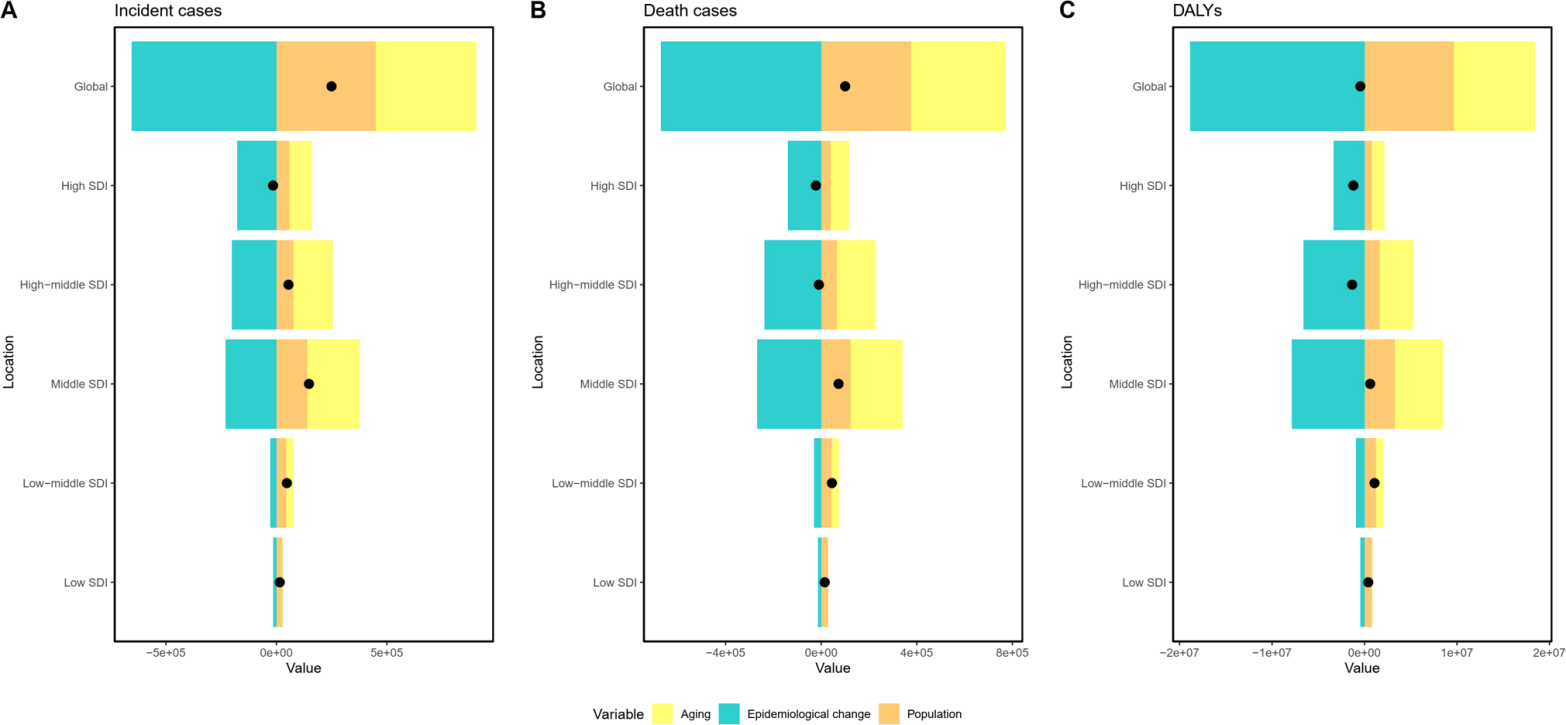
Visualization of decomposition analysis results. Black dots represent the overall changes in disease burden due to aging, epidemiological changes, and population growth. **(A)** incident cases from 1990 to 2021; **(B)** death cases from 1990 to 2021; **(C)** DALYs from 1990 to 2021. For each component, an increase in the disease burden of GC related to that component is indicated by positive values, whereas a decrease is indicated by negative values. SDI, Socio-demographic Index; DALYs, disability-adjusted life years; GC, gastric cancer.

Over the past 30 years, global GC cases have increased significantly. The primary drivers of this increase were population aging and growth, while epidemiological trends exerted a negative effect on the increase in incident cases. This negative influence of epidemiological trends was observed across all SDI groups. Similarly, the number of deaths has increased over the past three decades, with decomposition analysis results aligning closely with those of incident cases. In contrast, DALYs have shown a decreasing trend, especially in high and high-middle SDI regions, largely due to changes in epidemiological trends. Detailed results of the decomposition analysis are presented in **Table 2**, **Figure 5**.

**Table 2:** Detailed results of the decomposition analysis.

Measure	Location	Overall difference	Change due to population-level determinants (% contribute to the total changes)		
			Aging	Population	Epidemiological change
Incident cases	Global	249333.18	455234.57 (182.58)	449521.01 (180.29)	-655422.41 (-262.87%)
	High SDI	-15139.38	105691.84 (-698.13)	56589.15 (-373.79)	-177420.37 (1171.91%)
	High-middle SDI	54582.83	180158.1 (330.06)	76340.69 (139.86)	-201915.96 (-369.93%)
	Middle SDI	147575.65	239282.14 (162.14)	138180.68 (93.63)	-229887.16 (-155.78%)
	Low-middle SDI	47309.84	30958.86 (65.44)	43166 (91.24)	-26815.02 (-56.68%)
	Low SDI	15054.26	1010.2 (6.71)	27624.06 (183.5)	-13579.99 (-90.21%)
Death cases	Global	100189.08	395918.87 (395.17)	375507.69 (374.8)	-671237.48 (-669.97%)
	High SDI	-22143.33	78676.85 (-355.31)	38350.69 (-173.19)	-139170.86 (628.5%)
	High-middle SDI	-9668.07	162054.14 (-1676.18)	65359.32 (-676.03)	-237081.53 (2452.21%)
	Middle SDI	72443.12	219275.16 (302.69)	121495.79 (167.71)	-268327.83 (-370.4%)
	Low-middle SDI	44857.94	31486.64 (70.19)	42508.21 (94.76)	-29136.91 (-64.95%)
	Low SDI	14781.52	1077.2 (7.29)	27615.74 (186.83)	-13911.42 (-94.11%)
DALYs	Global	-450658.89	8749991.28 (-1941.6)	9663490.07 (-2144.3)	-18864140.24 (4185.9%)
	High SDI	-1202018.13	1334112.24 (-110.99)	821544.19 (-68.35)	-3357674.56 (279.34%)
	High-middle SDI	-1339631.24	3586151.67 (-267.7)	1659202.16 (-123.86)	-6584985.06 (491.55%)
	Middle SDI	612121.14	5207943.71 (850.8)	3270564.16 (534.3)	-7866386.74 (-1285.1%)
	Low-middle SDI	1078320.43	798609.04 (74.06)	1220102.33 (113.15)	-940390.94 (-87.21%)
	Low SDI	403262.73	33792.8 (8.38)	821068.34 (203.61)	-451598.41 (-111.99%)

SDI, Socio-demographic Index; DALYs, disability-adjusted life years.

**Figure 6:**
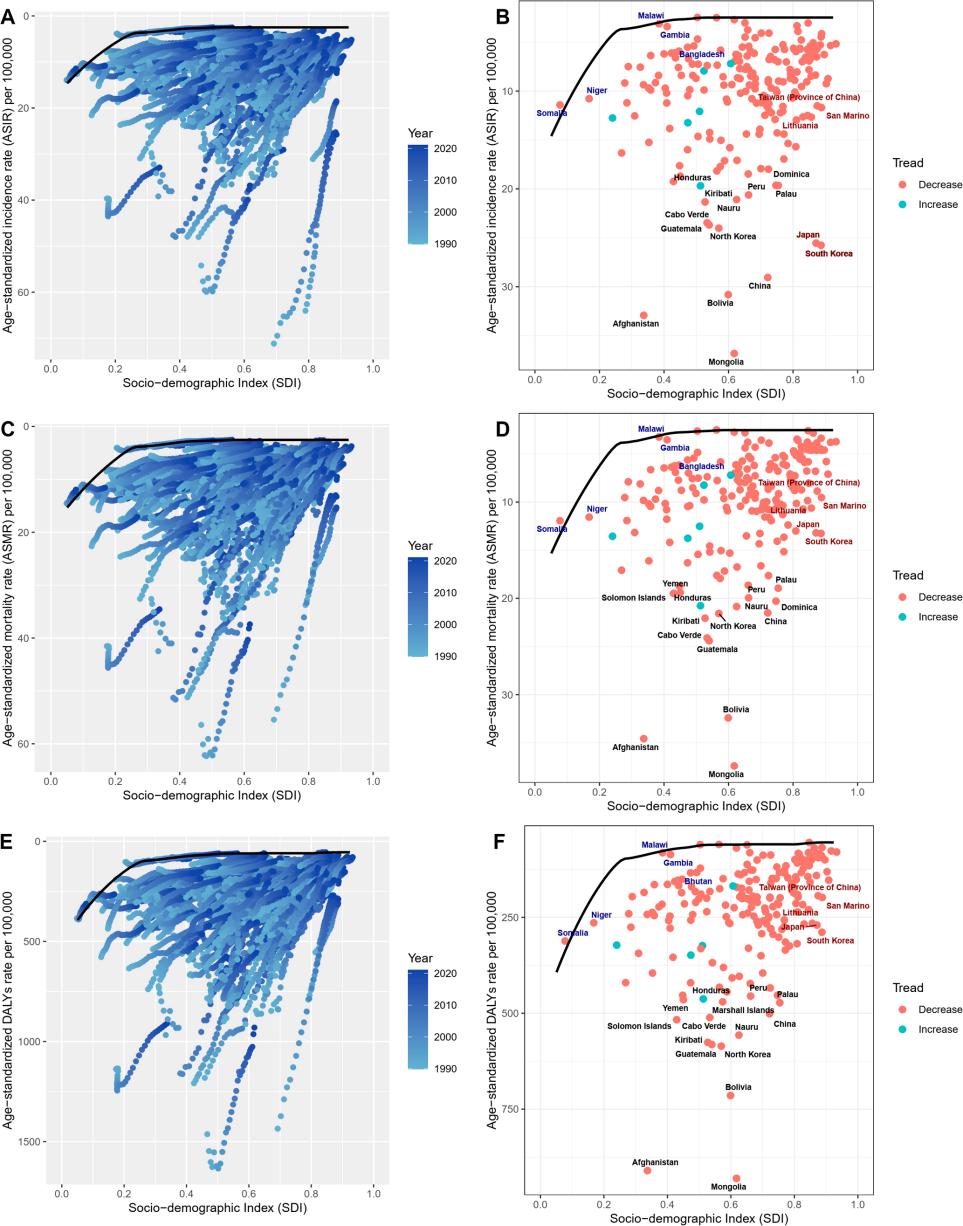
Visualization of frontier analysis results. **(A, B)** Frontier analysis for ASIR; **(C, D)** Frontier analysis for ASMR; **(E, F)** Frontier analysis for age-standardized DALYs rate. Black lines represent the lower limits of ASR achievable at different SDI levels, with points representing different countries and regions. The 15 countries and regions with the largest effective differences globally are labeled in black font, the 5 countries and regions with the smallest effective differences among low SDI countries are labeled in blue font, and the 5 countries and regions with the largest effective differences among high SDI countries are labeled in red font. In Figures **(A, C, E)**, the blue dots represent the ASRs of GC from 1990 to 2021, with darker shades indicating later years. In Figures **(B, D, F)**, the dots represent changes in GC ASR from 1990 to 2021. Blue dots indicate countries and territories where the ASR increased from 1990 to 2021, while red dots indicate countries and territories where the ASR decreased. ASIR, age-standardized incidence rate; SDI, Socio-demographic Index; ASMR, age-standardized mortality rate; DALYs, disability-adjusted life years; ASR, age-standardized rate; GC, gastric cancer.

Frontier analysis revealed that high SDI regions have significantly greater potential to reduce disease burden compared to low SDI regions. Notably, the 15 countries with the poorest performance are primarily located in middle and high-middle SDI regions. The distribution of 204 countries on the frontier analysis map shows a pattern of higher values in the middle and lower values on the edges, indicating larger disparities in disease burden among countries and regions within the middle and high-middle SDI groups. Detailed frontier analysis results are shown in **Supplementary Tables 8**-**10**; **Figure 6**.

### 3.6 Projections of GC ASRs from 2022 to 2040

**Figure 7:**
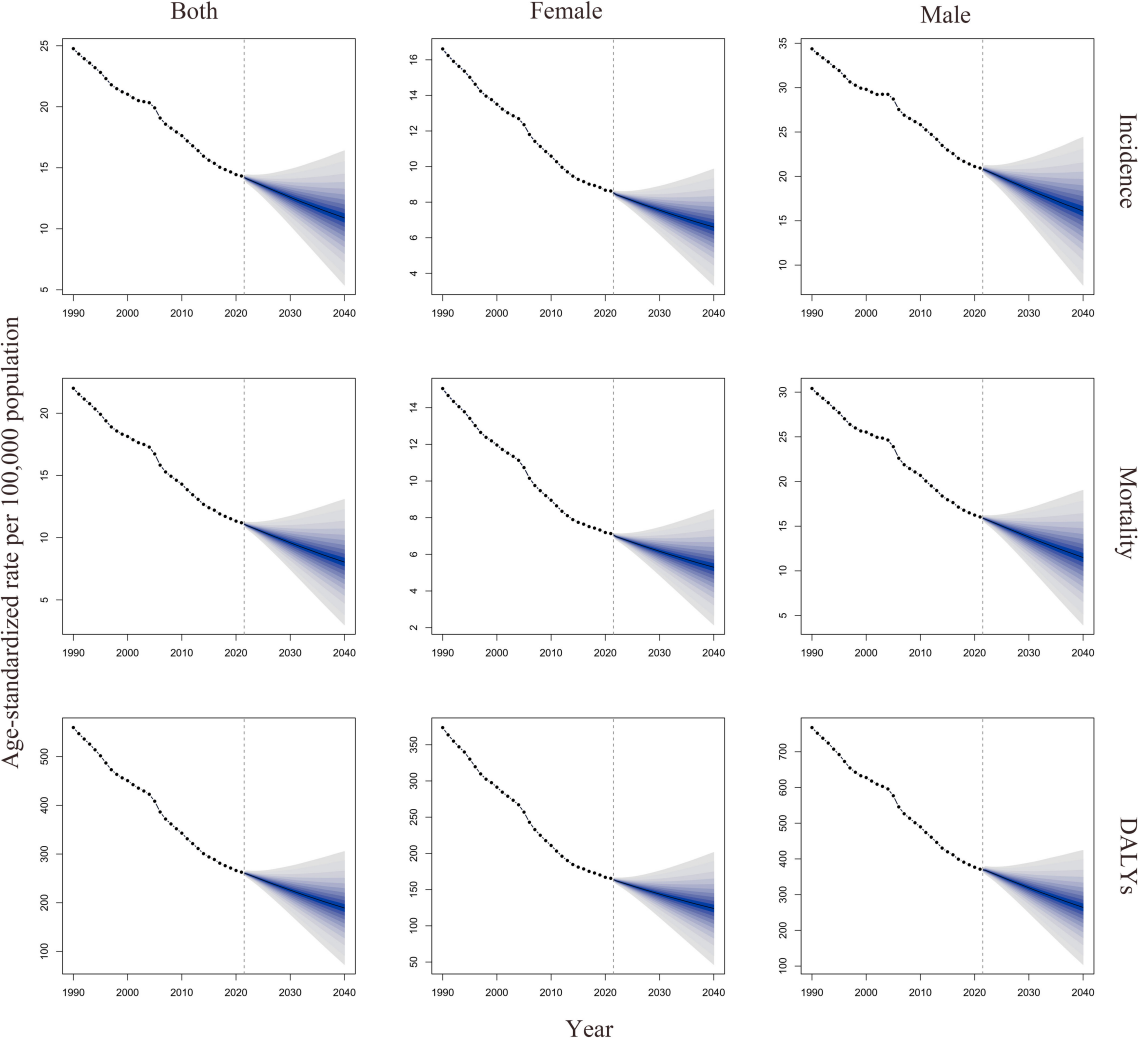
Visualization of BAPC predicted results. DALYs, disability-adjusted life years; BAPC, Bayesian Age-Period-Cohort model.

Using the BAPC model, the study projected the ASRs of GC up to 2040. The projections indicate that ASIR, ASMR, and age-standardized DALYs rates for both genders will continue to decline, with similar trends observed across these indicators. Detailed projection results from the BAPC model are provided in **Table 3**, **Figure 7**.

**Table 3:** Prediction of ASRs from 2022 to 2040.

Year	ASIR			ASMR			Age-standardized DALYs rate		
	Both	Female	Male	Both	Female	Male	Both	Female	Male
2022	14.09	8.42	20.69	10.98	6.96	15.78	258.98	162.16	368.03
2023	13.9	8.31	20.42	10.8	6.86	15.53	254.76	159.78	361.87
2024	13.72	8.2	20.15	10.63	6.76	15.28	250.52	157.43	355.66
2025	13.52	8.09	19.87	10.45	6.66	15.02	246.22	155.07	349.35
2026	13.33	7.97	19.6	10.28	6.56	14.77	241.98	152.73	343.12
2027	13.14	7.86	19.33	10.1	6.46	14.52	237.89	150.46	337.1
2028	12.96	7.76	19.07	9.93	6.36	14.27	233.9	148.25	331.2
2029	12.77	7.65	18.8	9.76	6.26	14.03	229.9	146.06	325.25
2030	12.58	7.55	18.53	9.59	6.17	13.78	225.83	143.86	319.2
2031	12.4	7.45	18.26	9.42	6.07	13.53	221.82	141.7	313.23
2032	12.22	7.34	18.01	9.26	5.98	13.3	217.99	139.6	307.5
2033	12.05	7.25	17.76	9.1	5.89	13.07	214.29	137.57	301.98
2034	11.88	7.15	17.52	8.95	5.8	12.83	210.6	135.55	296.46
2035	11.71	7.05	17.26	8.79	5.71	12.6	206.84	133.52	290.84
2036	11.53	6.96	17.01	8.63	5.63	12.37	203.12	131.53	285.24
2037	11.37	6.87	16.78	8.48	5.54	12.15	199.56	129.6	279.86
2038	11.21	6.78	16.55	8.33	5.46	11.93	196.13	127.71	274.69
2039	11.05	6.69	16.32	8.19	5.38	11.71	192.7	125.84	269.51
2040	10.89	6.6	16.08	8.04	5.3	11.49	189.19	123.96	264.21

ASRs, age-standardized rates; ASIR, age-standardized incidence rate; ASMR, age-standardized mortality rate; DALYs, disability-adjusted life years.

## 4 Discussion

This study provided comprehensive evidence on burden and trend of GC for the last three decades. The study revealed that despite the significant increase in the number of new cases and deaths, the ASIR, ASMR, and age-standardized DALYs rate have declined, with significant heterogeneity across different regions, SDI categories, and countries. Population growth and aging have contributed to the increase in new cases, deaths, and DALYs of GC, while changes in epidemiological trends have had a negative effect on these increases. According to projections, the ASIR, ASMR, and age-standardized DALYs rate of GC are expected to show a declining trend by 2024.

From 1990 to 2021, the number of new GC cases globally increased by 25.42%. Although the incidence rate of GC has declined in recent years, the effects of population growth and aging have offset this decline, resulting in a continued rise in new cases (23). This perspective has been demonstrated in the decomposition analysis of this study. East Asia remains a region with a high number of new GC cases, with China (mainland) and Japan leading in new cases, accounting for 57.78% of global new cases. This prevalence is closely related to the region’s adverse lifestyle factors and high Helicobacter pylori infection rates (24, 25). Specifically, China accounts for 49.73% of global new cases, marking a 1.43% increase compared to 2019 (26). This growth is not only related to the rapid population increase but may also be associated with improvements in the region’s economic level, healthcare conditions, and screening capabilities. To reduce the number of GC cases, future efforts should focus on decreasing the intake of high-salt foods, preserved foods, alcohol, and tobacco, as well as implementing Helicobacter pylori screening and treatment to lower infection rates. In Japan, radiographic screening programs for GC have been actively promoted since the 1960s, and regular screening is now conducted for individuals over 40 years old, resulting in higher GC diagnosis rates, which may contribute to the higher number of patients (27). India ranks third globally in new GC cases, with high Helicobacter pylori infection rates (28). In some regions, a preference for pickled or smoked meats is a significant factor contributing to its high GC incidence (29). However, India currently lacks effective population-based GC screening methods, leading to a high risk of missed diagnoses (28). The actual number of new GC cases could be higher than the estimates reported in the study.

Between 1990 and 2021, the ASIR of GC has decreased in most countries worldwide. This decline is strongly linked to improved hygiene, dietary changes (such as increased fruit and vegetable intake and reduced salt consumption), widespread use of antibiotics, and a lower prevalence of Helicobacter pylori infections (30). In South Korea, the preference for foods like kimchi and salted fish contributes to a high number of new GC cases, with its ASIR ranking fifth globally (31). However, our study found that South Korea has experienced the largest decrease in ASIR, likely due not only to reduced intake of kimchi and salted fish but also to its longstanding early screening programs (31, 32). Early screening helps identify precancerous lesions and enables timely intervention, effectively reducing the ASIR of GC. Additionally, Mongolia not only has the highest ASIR but also the largest increase in ASIR from 1990 to 2021. This may be related to the country’s high smoking rate, high Helicobacter pylori infection rate, low fruit and vegetable intake, and high-salt diet (24).

From 1990 to 2021, the global number of GC deaths showed an upward trend, with population growth and aging playing significant roles. However, the ASMR declined in most countries and territories, indicating significant progress in global GC prevention and treatment efforts. China has the highest number of GC deaths in the world, and GC is the second leading cause of cancer-related death in the country, posing a serious threat to public health (33). This study found that, although the number of deaths slightly increased by 19.0% from 1990 to 2021, the ASMR significantly decreased (AAPC: -2.44, 95% CI: -2.62 to -2.26). Despite the lack of nationwide GC screening in China, since 2006, endoscopic screening has been implemented in high-risk regions (25). Early screening for GC has significantly reduced mortality through early diagnosis, with the five-year survival rate increasing from 63.7% to 89% (25, 34, 35). Additionally, this progress is attributed to the improvement in standardized treatment and multidisciplinary management of GC over the past few decades. For example, postoperative adjuvant chemotherapy has improved the overall survival rate by 32% and the recurrence-free survival rate by 51% (25). In countries like South Korea, Japan, Singapore, and some high-SDI European regions, where more resources have been invested, early diagnosis, as well as Helicobacter pylori screening and eradication, have shown even more significant results, leading to a greater reduction in ASMR. In contrast, in regions with low SDI such as Africa, South Asia, and Latin America, although ASMR has also declined, the decrease has been relatively modest. Therefore, we believe that early screening and timely, standardized treatment are crucial strategies for reducing GC mortality. However, considering the lack of sufficient economic resources in underdeveloped regions for large-scale screening, we recommend that these areas adopt a high-risk regional screening model combined with low-cost methods to reduce screening costs.

From 1990 to 2021, global GC-related DALYs and age-standardized DALYs rate have shown a continuous decline. Decomposition analysis indicates that the primary driver of this decline is improvements in epidemiological trends, which have outweighed the rising effects of population growth and aging. The top four countries in DALYs are China, India, Japan, and Russia, which is closely linked to their large populations and high GC incidence. The decline in age-standardized DALYs rate is similar to the change in ASMR, largely attributed to the widespread use of GC screening and advances in comprehensive treatment for GC.

Although significant progress has been made in the prevention and treatment of GC worldwide, substantial disparities still exist across countries and regions. In 2021, China (mainland) had the highest number of new cases, deaths, and DALYs of GC globally, accounting for about half of the global total. It is projected that the number of GC cases in China will continue to rise in the future. This phenomenon is not only related to its large population base but is also closely associated with the accelerated aging process in recent years (36). Although GC screening has been implemented in high-risk areas, there is an expectation to further promote large-scale screening nationwide in the future (25). Additionally, reducing the intake of tobacco, alcohol, and high-salt diets, and actively lowering Helicobacter pylori infection rates, are key to prevention and control. Mongolia, despite its smaller population, has the highest GC ASR worldwide, likely related to the region’s high smoking rate (37). South Korea stands out as a country with a high ASIR but leads globally in the reduction of ASMR and age-standardized DALYs rate, a success closely tied to its comprehensive GC screening system (27).

From **Tables 1**–**3**, which shows data for the five SDI regions, it is evident that the ASIR, ASMR, and age-standardized DALYs rate are significantly higher in the high SDI, high-middle SDI, and middle SDI groups compared to the low SDI and low-middle SDI groups. We believe that this difference is not only due to the higher prevalence of risk factors for GC, such as smoking and alcohol consumption, in developed regions, but also because developed countries have more comprehensive GC screening systems, leading to lower rates of missed diagnoses. Additionally, this study found that from 1990 to 2021, the ASIR, ASMR, and age-standardized DALYs rate for GC have decreased more significantly in higher SDI countries, a trend consistent with previous research (26). Higher SDI countries generally have more advanced public health policies, which improve healthcare conditions and significantly reduce the infection rate of high-risk factors for GC, such as Helicobacter pylori infection, through screening and treatment. Moreover, the early screening systems for GC in high SDI regions are more robust, allowing for earlier detection of precancerous lesions and timely intervention, which helps to reduce GC incidence. These regions are also more likely to diagnose GC at an earlier stage and provide treatment promptly, leading to lower ASMR and age-standardized DALYs rate. Furthermore, patients in high SDI regions have greater access to the benefits brought by the rapid advancement of comprehensive treatment options for GC in recent years, further contributing to the reduction of ASMR and age-standardized DALYs rate in these regions.

From the perspective of gender differences, this study found that the incident cases, deaths, DALYs, and ASR of GC are significantly higher in males than in females. This may be related to lifestyle and physiological differences. A study reported that the smoking rate for males was 32.7%, compared to 6.62% for females, indicating that the smoking rate among males is significantly higher than that of females (38). Research indicates that harmful substances in tobacco smoke can lead to peptic ulcers, chronic inflammation, and mucosal cell proliferation, thereby increasing the risk of GC (39–42). Despite measures taken by some countries and regions to reduce smoking rates, the global smoking burden remains heavy, necessitating more effective tobacco control policies in the future (43). Additionally, males have a higher rate of alcohol consumption than females. A meta-analysis of 106 epidemiological studies, involving over 25 million participants, showed a significant association between alcohol consumption and GC, consistent with findings from multiple meta-analyses, explaining the incidence rate differences between genders (44, 45). Additionally, some studies have reported that estrogen can have a protective effect against various digestive system diseases, including GC (46). This could become a new direction for future treatment and prevention.

Decomposition analysis shows that population aging and growth are the main factors contributing to the increased burden of GC, particularly in countries like China and Japan, which have large populations and are experiencing aging demographics (47, 48). These countries will face significant challenges in reducing the GC burden in the future. According to frontier analysis, higher SDI regions have greater potential for reducing ASR. These regions should further improve their social development, enhance disease screening systems, and increase early diagnosis rates. Additionally, these regions should address unhealthy lifestyle habits, such as high intake of spicy and salty foods, and improve Helicobacter pylori treatment systems.

This study also compared the estimated data from GBD 2021 with the results from GLOBOCAN 2022 (3). GLOBOCAN 2022 reported 968,350 new GC cases in 2022, which is lower than the 1,230,233 cases reported by GBD 2021. Additionally, GLOBOCAN reported 659,853 deaths from GC, also lower than the 954,374 deaths reported by GBD 2021. Although China, India, and Japan are ranked among the top countries in both studies for new GC cases and deaths, the specific estimated values differ. We believe this discrepancy may be related to differences in the data sources and the number of countries included in the two studies. Specifically, GBD 2021 includes data from 204 countries and territories, while GLOBOCAN 2022 includes only 185 countries and territories. Compared to the estimates from GBD 2019, our study found a decrease in the ASIR, ASMR, and age-standardized DALYs rate for GC. We believe this decline is closely related to the global reduction in Helicobacter pylori infection rates, improvements in cancer screening systems, and advances in healthcare.

This study comprehensively explored global trends in GC burden using GBD 2021 data and projected the burden for 2040. However, the study has limitations. First, it analyzed overall GC without subgroup analysis, despite heterogeneity among different types of GC. Secondly, this study is a retrospective cross-sectional study, and the accuracy of the results largely depends on the reliability of the data used. However, there are significant differences in diagnostic practices, data recording, and healthcare accessibility across regions. In some areas, due to limited healthcare resources, comprehensive cancer registration and diagnosis may not be feasible, which could affect the accuracy of the estimates. In regions with insufficient data, the GBD study often relies on statistical models for estimation. While these models can partially compensate for missing data, they may still introduce biases. Furthermore, the BAPC model makes future predictions based on existing data trends, but it struggles to fully account for changes in external factors, such as advancements in medical technology, innovations in treatment methods, and adjustments in global health policies. These unpredictable factors could significantly impact the future disease burden, yet the model has limitations in capturing the potential effects of these changes on trends. Future research could enhance predictive accuracy by integrating multiple modeling approaches and incorporating more real-time data, better addressing the potential impact of changes in the medical and policy environment on disease burden.

There are several areas worth exploring and pursuing. First, establishing more accurate and reliable data collection mechanisms is crucial. Such mechanisms will ensure data completeness and accuracy and enable real-time publication of disease burden data. This will help policymakers promptly grasp trends and develop more targeted policy measures. Second, artificial intelligence can be introduced to analyze global disease burden, utilizing advanced algorithms and models to enhance the efficiency and precision of data analysis. Lastly, future GBD research requires multidisciplinary collaboration, bringing together experts in epidemiology, statistics, data science, public health, and clinical medicine. This cross-disciplinary approach will significantly enhance the breadth and depth of research and foster the development of innovative solutions.

## 5 Conclusion

This study explores the changing trends in the global burden of GC from 1990 to 2021. The findings indicate that although the ASRs of GC have decreased over this period, the number of cases and deaths has increased. The burden of GC is higher in men compared to women. With increasing SDI, the ASRs exhibit a fluctuating trend, with middle and high-middle SDI regions experiencing higher rates. According to projections, the ASRs of GC are expected to decline by 2040.

## Data Availability

The original contributions presented in the study are included in the article/**Supplementary Material**. Further inquiries can be directed to the corresponding author.
