# Supplementary material for: Global, regional, and national trends in gastric cancer burden: 1990-2021 and projections to 2040
Source: Front Oncol. 2024 Dec 12;14:1468488. doi: 10.3389/fonc.2024.1468488 (PMC11669584; doi:10.3389/fonc.2024.1468488)
Supplement: Supplementary file 1 [file DataSheet1.docx]

**
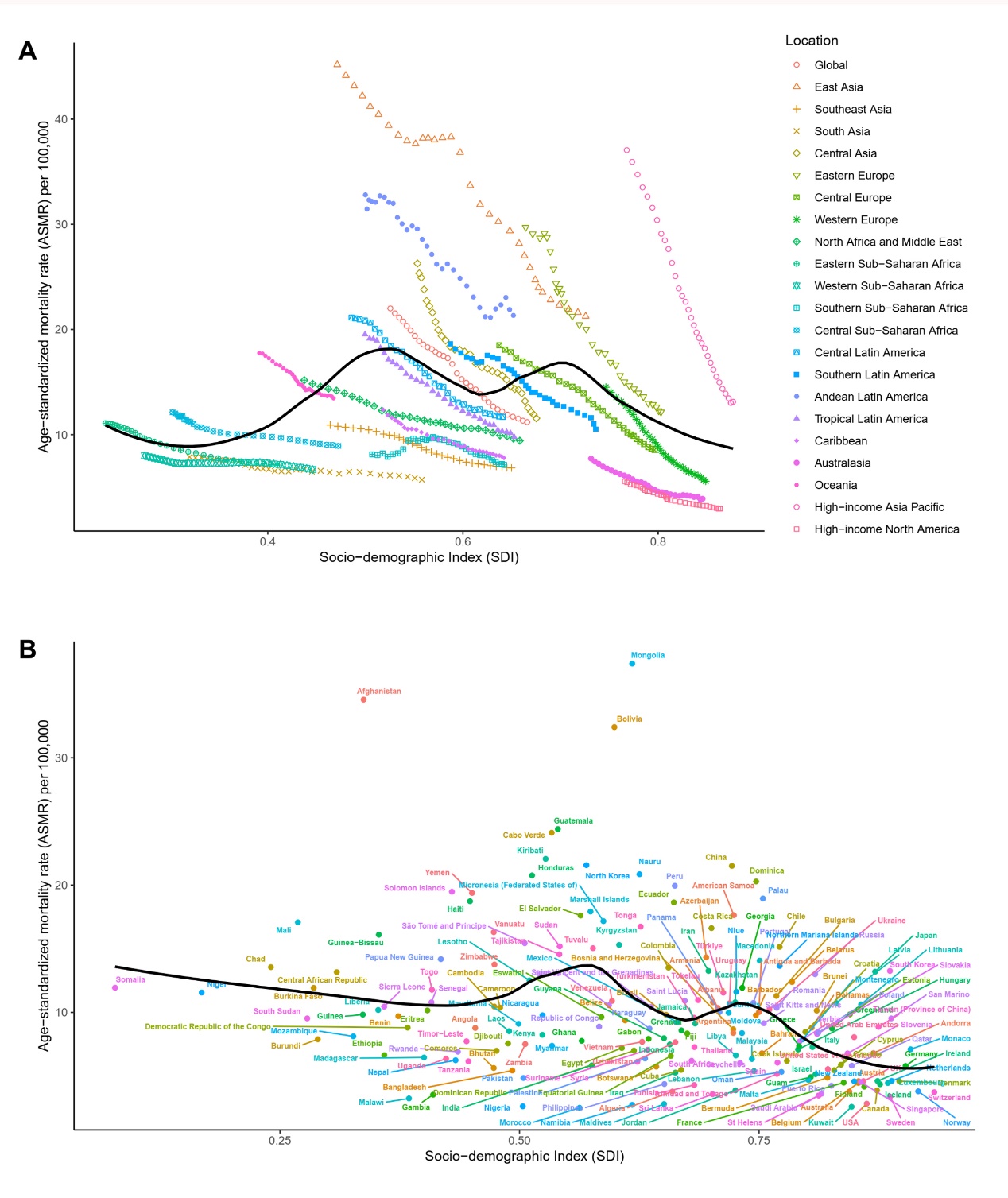
**

**Supplementary Figure 1:** Distribution of ASMR for GC across different SDI levels. A. Global and 21 GBD regions from 1990 to 2021; B. 204 countries and territories in 2021. ASMR, age-standardized mortality rate; SDI, Socio-demographic Index; GC, gastric cancer; GBD, Global Burden of Diseases, Injuries, and Risk Factors Study.


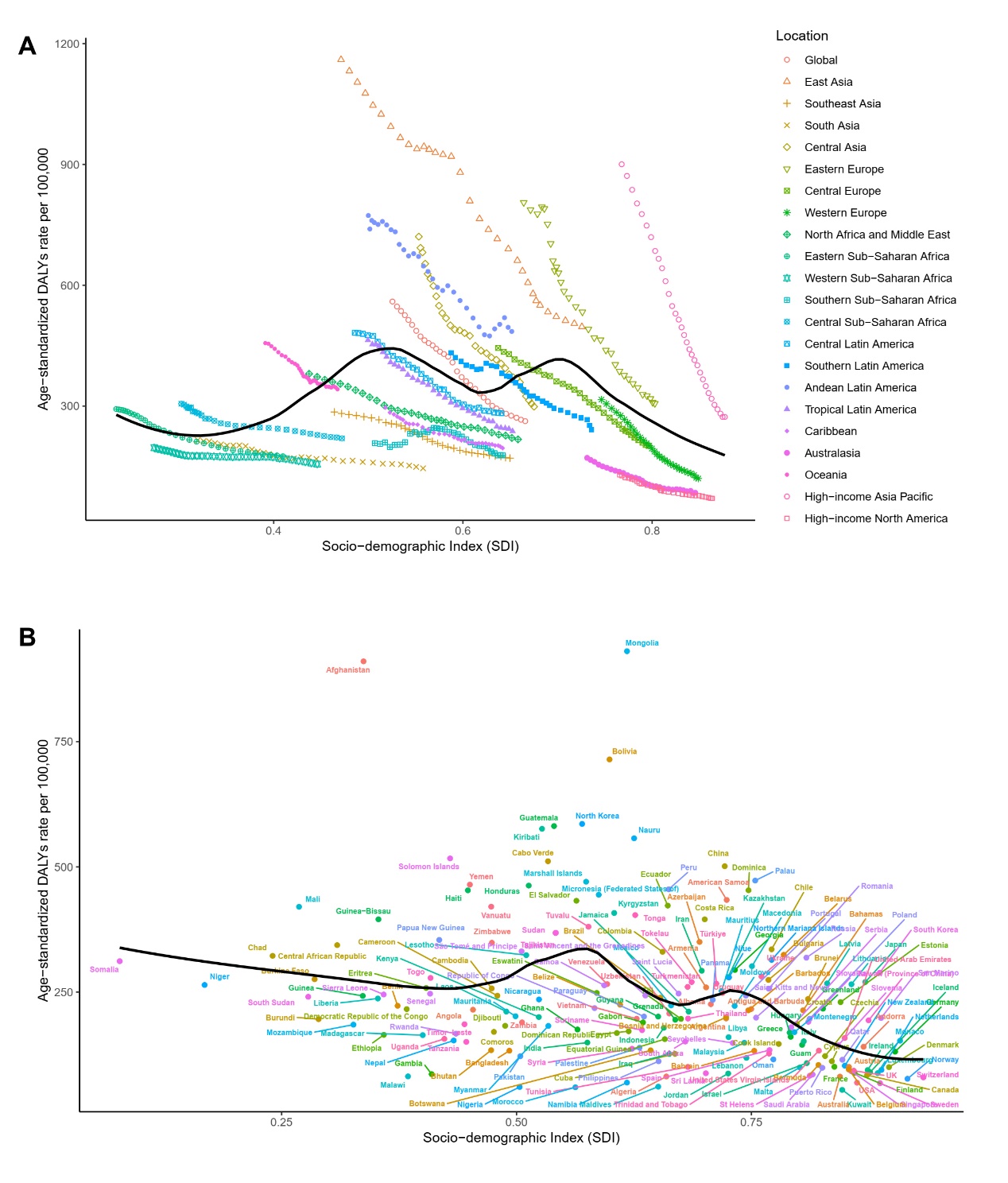


**Supplementary Figure 2:** Distribution of age-standardized DALYs rate for GC across different SDI levels. A. Global and 21 GBD regions from 1990 to 2021; B. 204 countries and territories in 2021. DALYs, disability-adjusted life years; SDI, Socio-demographic Index; GC, gastric cancer; GBD, Global Burden of Diseases, Injuries, and Risk Factors Study.

**
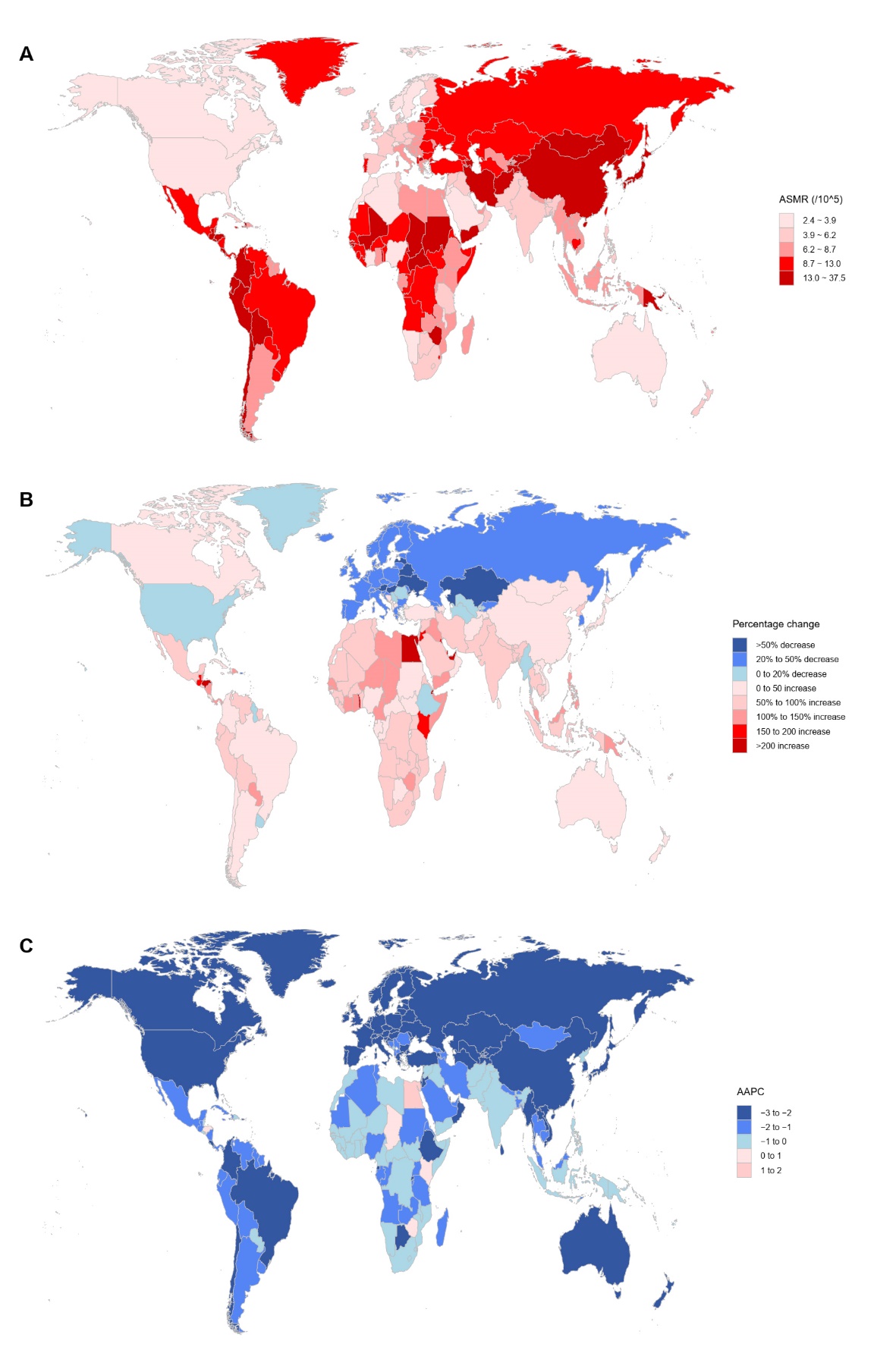
**

**Supplementary Figure 3:** Analysis of GC mortality using multiple indicators in 204 countries and territories. A. ASMR per 100,000 population in 2021; B. Percentage change in the number of Death cases from 1990 to 2021; C. AAPC from 1990 to 2021. ASMR, age-standardized mortality rate; AAPC, average annual percentage change; GC, gastric cancer.


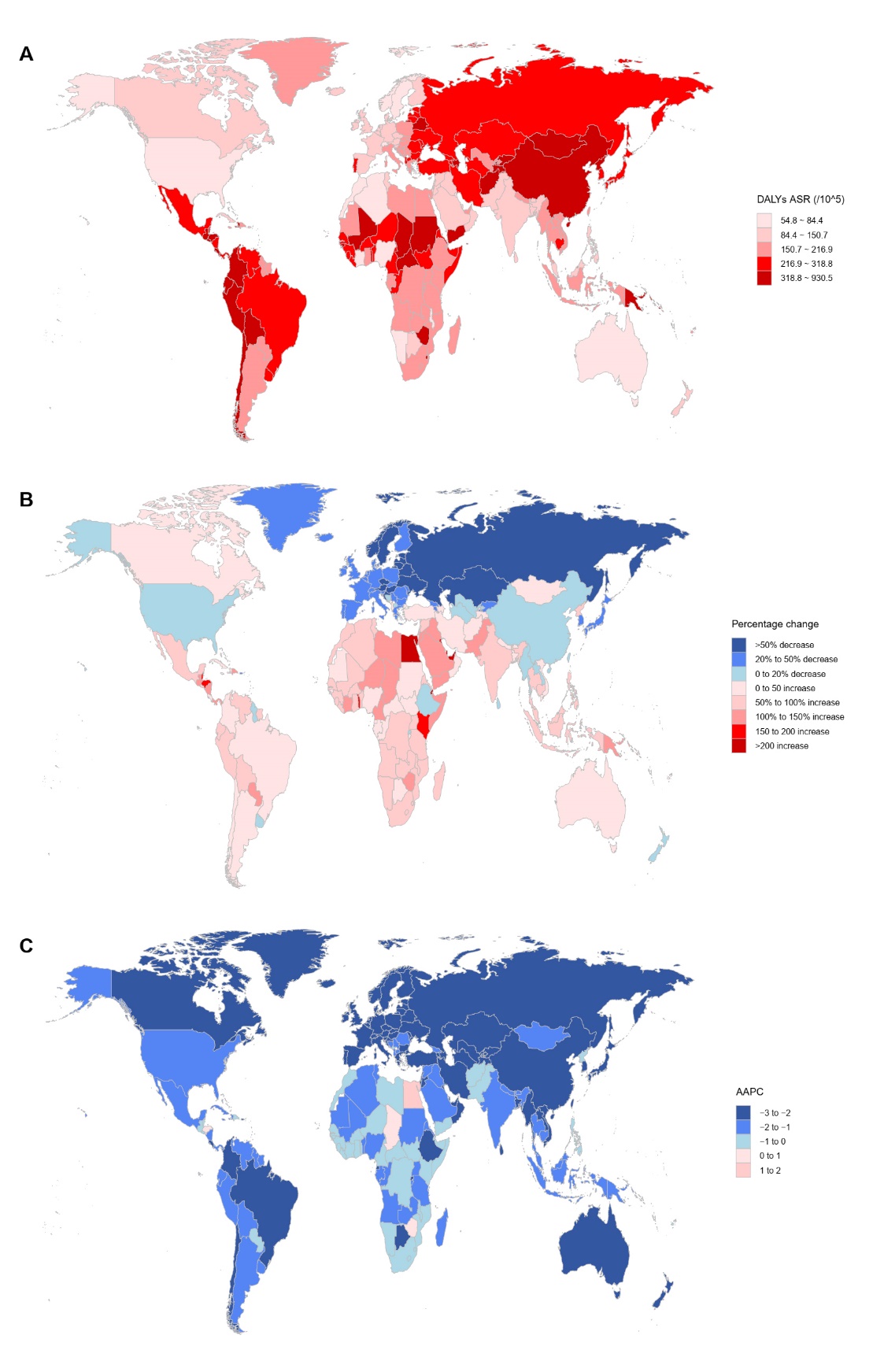


**Supplementary Figure 4:** Analysis of GC DALYs using multiple indicators in 204 countries and territories. A. Age-standardized DALYs rate per 100,000 population in 2021; B. Percentage change in the number of DALYs from 1990 to 2021; C. AAPC from 1990 to 2021. DALYs, disability-adjusted life years; ASR, age-standardized rate; AAPC, average annual percentage change; GC, gastric cancer.


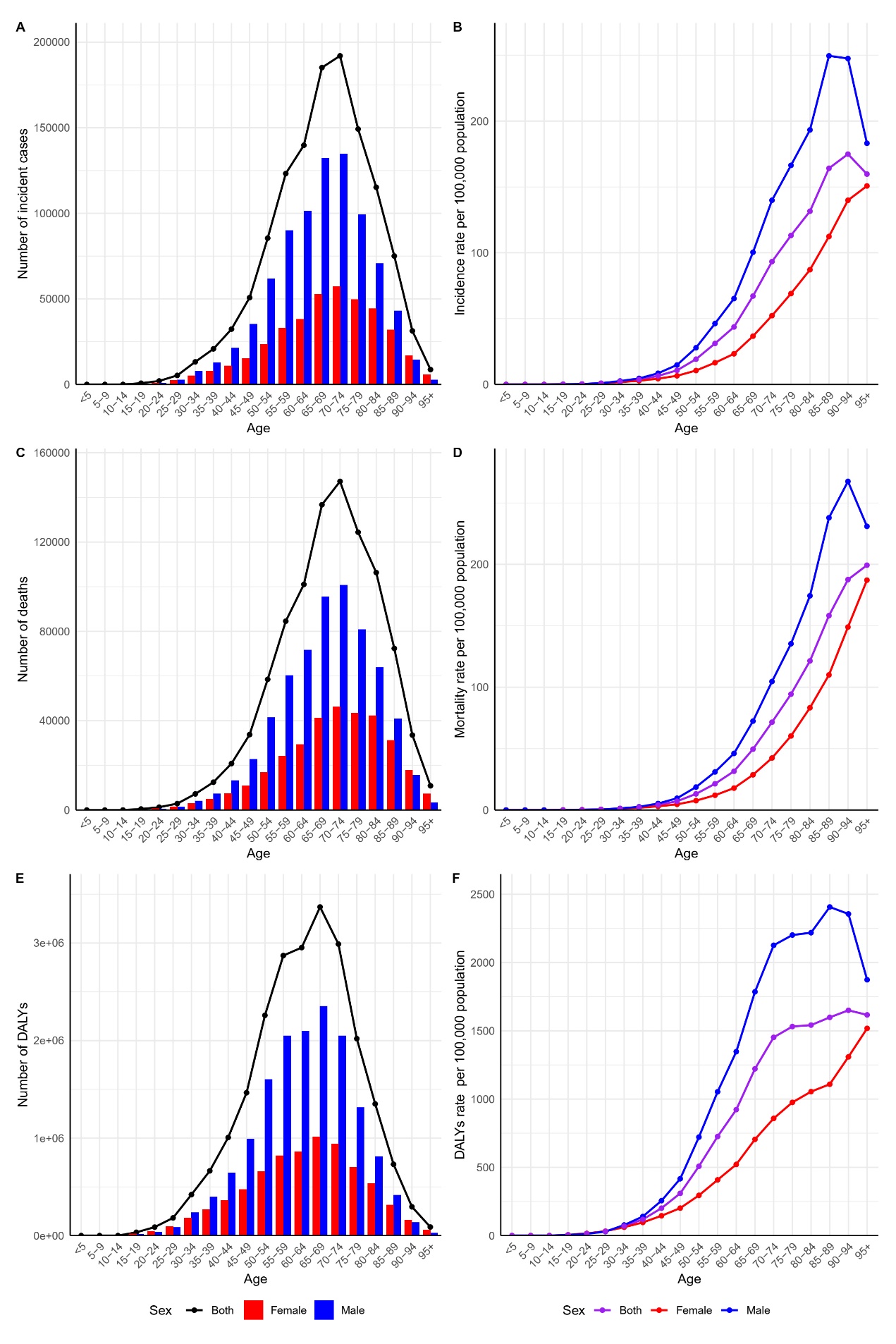


**Supplementary Figure 5:** Global burden of GC in 2021, stratified by sex and age. GC, gastric cancer.
